# Predictors of smoking among primary and secondary school students in Botswana

**DOI:** 10.1371/journal.pone.0175640

**Published:** 2017-04-17

**Authors:** Bontle Mbongwe, Roy Tapera, Nthabiseng Phaladze, Andrew Lord, Nicola M. Zetola

**Affiliations:** 1 Department of Environmental Health, University of Botswana, Gaborone, Botswana; 2 School of Nursing, University of Botswana, Gaborone, Botswana; 3 Good Business, London, United Kingdom; 4 Department of Radiation Oncology, University of Pennsylvania, Philadelphia, United States of America; National Yang-Ming University, TAIWAN

## Abstract

This study seeks to determine the prevalence and risk factors for smoking among students aged 12–18 years in two cities in Botswana. Using a sample of 2554 students we adapted the Global Youth Tobacco Survey (GYTS) methodology to assess students' smoking practices, knowledge, beliefs and attitudes. Logistic regression models were used to evaluate factors associated with smoking. The results revealed that 10% of students were current tobacco smokers with 29% reporting having tried smoking. Self-image and acceptance by peers were the strongest predictors of smoking overall (adjusted Odds Ratio [aOR]: 3.13, 95%, Confidence Interval [CI]: 2.67–3.66). Intention to smoke or to continue to smoke and perceived norms in conformity with smoking were also independently associated with smoking (aOR: 1.81, 95% CI: 167–2.11 and aOR: 1.31, 95% CI: 1.10–1.57, respectively). Perceived prevalence and exposure to smoking by peers and family and access to tobacco products was stronger among females (aOR: 1.69, 95% CI: 1.52–1.91) compared to males (aOR: 0.93, 95% CI: 0.70–1.24). Our results indicate that anti-tobacco interventions in Botswana should prioritize intra-personal factors associated with smoking. Our findings also suggest that different interventions targeting male and female students should be explored.

## Introduction

Tobacco smoking is the main preventable risk factor for a wide variety of medical conditions and one of the major public health problems worldwide. The magnitude of the problem is increasing in low-and middle-income countries, where the tobacco industry has shifted its attention [1]. Mortality due to tobacco smoking has been estimated at over 6 million deaths annually[2]. The World Health Organization (WHO) predicts that, if current patterns of tobacco consumption continue, more than 500 million people alive today will be killed by tobacco by 2030 [3]. This impact is not only due to morbidity and mortality, but also attributed to the social and economic cost of smoking which are more pronounced in resource-limited settings [4].

Evidence from sub-Saharan Africa shows a variation of 20–60% smoking prevalence among men and an increasing annual cigarette consumption rates for both men and women [5]. Additionally, there is compelling evidence from the WHO, that tobacco use has doubled in the last four decades particularly among the youth and the poor[6]. The increase in tobacco use is due to, among others, aggressive tobacco industry marketing of tobacco products to adolescents and young adults in low and middle-income countries [7–10] and early initiation of smoking [11, 12]. Current tobacco products use among adolescents aged 13 to 15 years vary from 5–10% [13]. Overall current cigarette smoking among 13–15 year olds in Botswana is 7% [14]. Most adult smokers in developed and developing countries report taking up the habit of smoking between the ages of 12 and 15 [15, 16] and one in four teenagers have become regular smokers by age 15 [17]. However, the prevalence and risk factors associated with tobacco smoking varies greatly from one country to the other [13]. In countries such as the United States, 11.1% of adolescents initiated smoking at age 10 years or younger [18] and the majority of students 16 years of age and older (60.4%) reported ever having smoked a whole cigarette. In a similar study conducted in India [19], nearly 70% of boys and 80% of girls ≤ 15 years of age initiated the habit of tobacco consumption before the age of 11 years. There is mounting evidence that adolescents who experience smoking initiation before age 14 also use alcohol and other drugs more frequently [20, 21]. Whilst these linkages are not yet established in Botswana, research from the Seychelles [21],a Southern African Development Community (SADC) member state like Botswana, shows that adolescent smokers were 2 times more likely than non-smokers to drink and nine times more likely to use cannabis.

Compared to other areas of the world, there is a paucity of data regarding the prevalence and risk factors for youth smoking in sub-Saharan Africa (SSA) [16]. This gap in knowledge is further compounded by the high burden of Human Immunodeficiency Virus/ Acquired Immunodeficiency Syndrome (HIV/AIDS) in the region. Due to increased access to antiretroviral therapy, people living with HIV are living longer and experiencing a significant increased incidence and prevalence of chronic diseases, many of which are associated with tobacco use [22–24].

These observations call for urgent interventions to prevent smoking initiation by adolescents. This study seeks to determine the prevalence of smoking and to identify the risk factors associated with smoking among primary and secondary school students in the two largest cities in Botswana.

## Materials and methods

### Study design

The study utilized a cross-sectional design and it was carried out in January 2014.

### Population and setting

All primary, junior and senior secondary school students from Gaborone and Francistown, the 2 largest cities in Botswana, were eligible for the study.

### Sampling

Sampling for this survey followed the GYTS sampling strategy [25, 26]. In brief, the sampling was conducted from a list of schools provided by the Ministry of Education and Skills Development to participate in the survey. The probability of selecting a school was proportional to the number of students enrolled in the specified grades. The survey used a two-stage, cluster-sample design to produce a representative sample of students aged 12–18 years attending public and private schools. Seventy-five (75) schools made up of 22 primary, 26 Junior secondary and 27 senior secondary schools from Gaborone and Francistown were randomly selected for inclusion in the survey. Specific classes for each selected school were then randomly selected. All students from the selected classes were eligible to participate in the survey.

### Primary outcome

Our study was aimed at determining the prevalence and risk factors for current smoking among students in Botswana. Current smoking was defined by self-report of having smoked a cigarette for >1 day during the preceding 30 days.

### Secondary outcomes

Our two secondary outcomes were 1) lifetime prevalence of smoking, defined as having ever smoked a cigarette; and 2) current and lifetime use of tobacco products other than cigarettes (which included chewing tobacco, snuff, dip, cigars, little cigars, pipes, or shisha)

### Questionnaires and measurements

Socio-demographic data included age, sex and study level. Study level was defined as: primary (standards 6 and 7), junior secondary (forms 1–3) and senior secondary (forms 4–6). The final questionnaire was aimed at determining intra-personal, cultural and/or environmental, and social or normative risk factors associated with smoking. Instrument development was based on focus groups conducted in Botswana. We first adapted and expanded the Global Youth Tobacco Survey (GYTS) to assess factors associated with smoking [26]. GYTS is an anonymous and confidential self-administered questionnaire designed for school-based surveys. Additional questions regarding locally relevant tobacco promoting and restraining factors were included. For every item, Likert-type scale questions were asked about how ‘negative’ the factor was considered by the student, ranging from 0 (not at all) to 4 (very much). The following 6 domains were included within the three thematic areas namely intrapersonal, social and normative, and culture and environment. The intrapersonal thematic area included questions regarding personal and behavioural factors as well as current behaviour relative to tobacco use. Three domains were included in this section: 1) Knowledge of the short and long-term health consequences of using tobacco; 2) Influence of peer groups and behavioural skills, subjective expected utility of tobacco use, and self-esteem and behavioural control; and, 3) Intentions to smoke and smoking status. The social and normative thematic area sought to determine the influence of the large social environment and the way society defines whether, when, and for whom smoking is acceptable. Whilst the dependent vatiable was smoking, Two domains for independent variables were included namely: 1) Perceived prevalence, perceived norms conformity, exposure to smoking by peers and family and access to tobacco products; and, 2) Exposure to mass media advertisement. We combined smoking by peers, siblings, and significant others into one domain. The final thematic area, culture and environment: included questions that sought to determine the effect of environmental factors (including acceptability and availability of tobacco products), communications in the mass media, interpersonal variables, and perceived environmental variables. These questions were grouped into a single domain. For internal consistency and scale reliability of the domains, we computed cronbach's alpha values (S1 Table).

### Pretesting of the questionnaire

The questionnaire was written in English and translated into Setswana (the local language) and back translated (S2 Table). Questionnaires were pre-tested in a pilot study using focus group discussions to assure internal and external validity. The results of the pilot study revealed that there was no ambiguity, redundancy and that language usage was appropriate for the target group. Prior to the pretesting, field assistants and study supervisors were trained on the methodology of the survey and familiarized with ethics requirements of the study. During the pilot study and the actual survey, students were allowed to complete the questionnaire in the language of their preference. However, all students completed the English questionnaire both in the pilot and actual survey implementation. Data were collected using anonymous self-administered questionnaire.

### Data management and analysis

Descriptive statistics were computed to describe all variables. Univariate and multivariate logistic regression models were used to evaluate domains associated with the primary and secondary outcomes. Scores for each domain were divided in tertiles and treated as ordinal variables for analysis. The lowest tertile was used as the reference category. Before fitting the model, we used factor extraction with varimax rotation to confirm how well the risks and benefits items loaded on their respective categories. The dimensionality of the domains was examined with correlation analysis and with factor analysis (principal axis factoring). The reliability of the scale was estimated with Cronbach’s α. All demographics and the 6 domains were included as variables for the multiple logistic regression analysis. Secondary models for male and female students were also fitted. We set the significance level at 5% alpha and excluded missing cases. Data were analyzed using STATA SE 13 software.

### Human subjects

This study was approved by the ethics committees of the University of Botswana and the Human Research Development Committee of the Botswana Ministry of Health.. The purpose of the study was well explained to students enrolled in the study. Written consent was obtained from heads of schools, parents, guardians and assent from the students. By signing the consent form and assent forms it indicated that the participants and their parents/guardians had agreed to participate in the study. There were no incentives given to participants. Participation in this study was voluntary and consent was sought from parents for students below 18 years and assent sought from students. For students above 18 years written consent was sought directly from students

## Results

### Description of study participants and smoking prevalence

Table 1 shows the characteristics of study participants. A total of 2554 (85%) out of 3000 students from 68 schools out of 75 participated in this study. The mean age of the participants was 15 years (Standard Deviation [SD] = 2.16) with an age range of 12–18 years. Males and females represented 1021 (42%) and 1411 (59%) of the respondents respectively.

**Table 1:** Characteristics of study population.

	Number of respondents (n)	Proportion (%)
**Gender**	**n = 2432**	
Male	1021	42
Female	1411	58
**Age in complete years**	**n = 2549**	
12 Years	484	19
13 Years	306	12
14 years	357	14
15 years	357	14
16 years	230	9
17 years	331	13
18 years	484	19
**Academic level**	**n = 2550**	
Standard 6	204	8
Standard 7	383	15
Form 1	357	14
Form 2	383	15
Form 3	357	14
Form 4	129	5
Form 5	537	21
Form 6	204	8
**School Category**	n = 68	
Primary	22	32
Junior secondary	24	35
Senior secondary	22	32
**City**		
Gaborone	1484	58
Francistown	1070	42

Two hundred and sixty one (10%) respondents were current tobacco smokers and 728 (29%) reported to have tried smoking cigarettes or any form of tobacco (Table 2). Overall, a higher proportion of girls (4.5%) compared to boys (3.0%) aged 12 years were current smokers but the difference was not statistically significant. Males (13%, n = 135) compared to females (9%, n = 126), reported current tobacco smoking. Current smoking was higher in the older age groups and was reported by 3.2% of males and 3.7% of females aged 12–13 years and 7.5% males and 7.1% of females aged 17–18 years (p = 0.001). From age 12 to 18, ever smoking prevalence increased from 6.8% to 23.3% and from 6.0% to 22.4% among males and females respectively. A similar increasing trend was observed in academic level and school category.

**Table 2:** Current smoker and ever-smoked associations with demographic variables.

Characteristic	Current Smoker		P value	Ever Smoked		P value
	Males n (%)	Females n (%)		Males n (%)	Females n (%)	
**Age**			**P = 0.72**			**P = 0.49**
**12 Years**	14(3.0)	21(4.5)		32(6.8)	28(6.0)	
**13 Years**	11(3.8)	8(2.7)		26(8.9)	23(7.9)	
**14 years**	14(4.1)	13(3.8)		36(10.6)	34(10.0)	
**15 years**	22(6.7)	18(5.5)		51(15.5)	47(14.2)	
**16 years**	13(6.3)	8(3.9)		38(18.2)	26(12.4)	
**17 years**	24(7.3)	27(8.2)		71(21.5)	70(21.2)	
**18 years**	37(8.5)	31(7.1)		104(23.3)	100(22.4)	
**Academic Level**			**P = 0.91**			**P = 0.03**
**Standard 6**	10(4.9%)	11(5.4)		20(9.9)	10(4.9)	
**Standard 7**	10(2.8%)	9(2.5)		15(4.1)	17(4.6)	
**Form 1**	16(4.7)	15(4.5)		37(11)	31(9.2)	
**Form 2**	21(5.8)	16(4.5)		52(14.4)	41(11.3)	
**Form 3**	18(5.3)	13(3.8)		60(17.5)	46(13.4)	
**Form 4**	10(8.9)	10(8.9)		24(21.4)	43(38.4)	
**Form 5**	28(5.7)	33(6.7)		108(21.4)	101(20.0)	
**Form 6**	21(11.2)	19(10.2)		42(22.7)	61(33.0)	
**School Category**			**P = 0.59**			**P < 0.01**
**Primary**	21(3.7)	20(3.5)		36(6.3)	26(4.6)	
**Junior Secondary**	62(5.9)	48(4.6)		153(14.6)	121(11.5)	
**Senior Secondary**	52(6.6)	58(7.4)		169(21.2)	181(22.7)	
**City**			**P = 0.16**			**P = 0.18**
**Gaborone**	84(6.0)	73(5.2)		208(14.8)	211(15.0)	
**Francistown**	51(5.1)	53(5.3)		150(14.9)	117(11.6)	

Overall, 9.4% (n = 239) reported the use of tobacco products other than cigarettes. These tobacco products were hubbly (36%), marijuana (26%), tobacco lotion (20%), snuff (7.9) and hashish (5%).

### Risk factors associated with smoking

Table 3 shows the univariate and multivariate analyses of risk factors associated with current smoking. Univariate analysis showed an association of current smoking with age. Nonetheless, the association was lost after controlling for other factors.

**Table 3:** Univariate and multivariate analysis of risk factors associated with smoking.

	Univariate analyses All		Multivariate analyses					
			All		Male		Female	
	OR	95% CI	aOR	95% CI	aOR	95% CI	aOR	95% CI
**Sex**	1.00	0.99–1.01	0.99	0.99–1.01	NA		NA	
**Age**	1.01	0.99–1.03	0.98	0.95–1.02	1.09	0.94–1.26	0.99	0.93–1.06
**School grade**	1.03	1.00–1.05	0.98	0.96–1.05	0.97	0.85–1.11	0.97	0.87–1.09
**Knowledge**	1.48	1.22–1.79	1.33	1.09–1.62	1.18	0.87–1.61	1.51	1.14–1.99
**Self-image and peer-pressure**	3.24	2.79–3.76	3.13	2.67–3.66	2.85	2.27–3.56	3.16	2.5–4.00
**Intentions to smoke**	1.43	1.22–1.66	1.81	1.67–2.11	1.24	0.96–1.60	1.05	0.81–1.37
**Perceived prevalence; exposure to smoking by peers & family & access to tobacco products**	1.42	1.13–1.89	1.31	1.10–1.57	0.93	0.70–1.24	1.69	1.52–1.91
**Exposure to media adverts**	1.34	1.11–1.60	1.19	1.01–1.40	1.06	0.81–1.39	1.55	1.18–2.03
**Culture and environment**	1.28	1.09–1.51	1.08	0.98–1.19	1.51	1.14–1.99	1.21	0.94–1.57

OR = Odds Ratio

aOR = adjusted Odds Ratio

CI = Confidence Interval

There was an association between knowledge of risks associated with tobacco use and smoking across both sexes (adjusted odds ratio [aOR]: 1.33, 95% Confidence Interval [CI]: 1.09–1.62); however, the effect size for this association was higher among females (aOR: 1.51, 95% CI: 1.14–1.99). Overall, perception of the effect of smoking over self-image and acceptance by peers were predictors for smoking (aOR: 3.13, 95% CI: 2.67–3.66). Intention to smoke or continue to smoke (aOR: 1.81, 95% CI: 1.67–2.11) and perceived norms conformity with smoking (aOR: 1.31, 95% CI: 1.10–1.57) were also independently associated with current smoking. After controlling for other factors, the association between smoking exposure to media advertisement remained significantly associated with smoking among girls (aOR: 1.5, 95% CI: 1.18–2.03). Perceived prevalence and exposure to smoking by peers and family and access to tobacco products was stronger among girls compared to boys (aOR:1.69, 95% CI: 1.52–1.91 and aOR: 0.93, 95% CI: 0.70–1.24 respectively).

## Discussion

Africa has been identified as the future epicenter of the global tobacco epidemic [27, 28]. Youth smoking data show critical warning signs that smoking prevalence among boys is higher than in other developing regions, and smoking prevalence among girls is higher compared to women. Our study confirms the severity of the problem in Botswana; as 10% of the students from the two major urban settings in the country were current smokers by self-report. Further, the magnitude of the problem is likely to be increasing since the proportion of students aged 13–15 who have tried smoking increased from 13.1% in 2002 [29] to 17.0% in 2008 and 29% in our study [30]. Our results are consistent with data from previous studies in sub-Saharan countries showing an increasing trend on smoking rates as well as early smoking initiation in adolescence [20], suggesting that increasing youth smoking is a major public health concern in the entire region.

The prevalence of smoking across all ages and education levels indicates that tobacco prevention programs must target adolescents of all ages. Furthermore, since a significant number of 12 year-old adolescents attending primary schools were already current smokers, tobacco prevention efforts might need to be started even before the children become adolescents. Contrary to prior reports from the region, in our study males and females showed similar prevalence of smoking, indicating that girls in Botswana might be more vulnerable compared to those from other countries [31]. The high number of girls aged 12 years who are current smokers is equally a cause for concern. There is a need to establish the reasons for this trend.

Tobacco smoking was found to be significantly higher among senior students as opposed to those in junior secondary school levels. Similar findings have been reported by other authors [32–34], showing the risk of tobacco consumption increasing with students' progression. The reasons behind such increased risk remain unclear and are likely to be complex. However, the increased self-report of stress and peer-pressure as a reason for smoking and the association between smoking and having seen someone smoking in films, videos and social media, point towards some of the factors that could be attributed to this trend. The relationship between cigarette marketing and identifiable adolescent needs, such as peer acceptance, rebelliousness, risk taking, and stress relief have been found in other studies [35]. In this study the odds of smoking were greatly increased by self-image misconceptions, perceived prevalence of smoking among peers and family smoking and students’ perception of smokers. This is consistent with other studies [36–38]. Our findings bring out key issues of concern. First, the perceived belief that peer smoking is highly prevalent may contribute to future smoking and the belief by adolescents that smoking is an acceptable behavior. This thinking is supported by our finding of the “intention to smoke” in this and other studies [39, 40] as a predictor of smoking. Second, many studies [37, 41–43] have shown that adolescent smoking is correlated with family smoking status. Evidence exists that family members who smoke not only create a positive norm for adolescents to smoke, but also make it easier for adolescents to access tobacco products and therefore easier to smoke [42, 44]. This calls for interventions to reduce parental smoking in order to de-normalize smoking among adolescents.

Bans on tobacco advertising, as outlined in the WHO Framework Convention on Tobacco Control (FCTC), could help stop the increase in tobacco use among adolescents, particularly girls. Our study shows that girls are more vulnerable to media advertisements than boys. In their study, Pierce et al. (1994) concluded that tobacco advertising campaigns targeting women, are associated with a major increase in smoking uptake specific to females younger than the legal age for purchasing tobacco products [45]. Botswana is a Party to the FCTC and Parties to the FCTC are encouraged to enact laws that have comprehensive bans on tobacco advertising and promotion (TAPS). Currently, point of sale advertising and indirect advertising in the form of attractive packaging of cigarettes meant to appeal to young girls such as “slims cigarettes” remain a challenge [46, 47]

Our study also highlights some of the complexities related to the implementation of successful preventive interventions. Students showed high awareness of the risks of smoking especially in relation to the significant role of smoking in lung cancer. However, this knowledge was not protective towards smoking. Further, we did not find any protective effect of having seen smoking prevention messages or messages on the harmful effects of smoking over tobacco smoking. In sum, these findings suggest that while current smoking prevention campaign may increase knowledge about the harmful effects of tobacco use, they are not successful in preventing youth smoking behavior. Conversely, there is need for the development of more effective smoking-prevention interventions targeting adolescents.

Culture and the environment had a higher effect on smoking among adolescent boys than girls. Perceived peer and social acceptability was a strong predictor of smoking among boys. This result is consistent with literature showing that adolescent smokers are more likely to perceive smoking as more accepted and normative [48].

## Study limitations

The findings in this study have the following limitations. First, these data apply only to adolescents aged 12–18 years who attended school in urban cities. This may limit the generalizability of the findings. Second, these data apply only to adolescents who were in school on the day of survey administration. Third, the data are all based on self-reports, possibly leading to response bias.

## Conclusions

Tobacco advertising, the intentions to smoke, and perceived peer and family smoking status were associated with smoking among adolescents in Botswana. Even more concerning is that having seen smoking prevention messages did not discourage adolescents from smoking. These findings suggest that the current health promotion interventions do not target the contemporary social and environmental contexts of adolescents in Botswana. Whilst adolescent girls are more sensitive to tobacco advertising and marketing, adolescent boys are more affected by cultural and environmental factors within their immediate environment. There is need to develop interventions such as peer education which will address the complexities of adolescent smoking and help them achieve self-image without feeling the pressure to smoke. To address parental influence on smoking behavior, it is necessary to initiate smoking cessation programs for parents and guardians. To the best of our knowledge, there are currently no smoking cessation programs in Botswana. Such interventions may reduce the number of adults smoking, therefore reducing the impact of parental smoking on adolescents.

## Supporting information

S1 Table Predictors of smoking: Domains and Cronbach’s Alphas. (DOCX)

S2 Table Pretested English questionnaire. (DOC)
