# Supplementary material for: Predictors of smoking among primary and secondary school students in Botswana
Source: PLoS One. 2017 Apr 17;12(4):e0175640. doi: 10.1371/journal.pone.0175640 (PMC5393585; doi:10.1371/journal.pone.0175640)
Supplement: S1 Table — (DOCX) [file pone.0175640.s001.docx]

**S1 Table: Predictors of Smoking: Domains and Cronbach’s Alphas**

| Measure | Chronbach’s Alpha |
| --- | --- |
| Intrapersonal: |  |
| Knowledge of the short- and long-term health consequences of using tobacco | 0.74 |
| Influence of peer groups and behavioral skills, subjective expected utility of tobacco use, and self-esteem and behavioral control | 0.82 |
| Intentions to smoke and smoking status. | 0.88 |
| Social and/or normative: |  |
| Perceived prevalence, perceived norms conformity, exposure to smoking by peers and family and access to tobacco products; | 0.71 |
| Exposure to mass media advertisement. | 0.83 |
| Cultural and/or environmental | 0.73 |
