# Supplementary material for: Predictors of smoking among primary and secondary school students in Botswana
Source: PLoS One. 2017 Apr 17;12(4):e0175640. doi: 10.1371/journal.pone.0175640 (PMC5393585; doi:10.1371/journal.pone.0175640)
Supplement: S2 Table — (DOC) [file pone.0175640.s002.doc]

| **Supervisor Code** |  |
| --- | --- |
| **Field Assistant Code** |  |
| **Date** | **Day/Month/Year** |

**Survey about cigarette smoking**

This survey asks you about how you feel about cigarette smoking. We promise that your answers will be kept secret and not shown to anyone outside the research team. Do not write your name on the form. It is very important to be as truthful as possible.

**First, we would like to know about your background**

***Note: Please circle the categories for the correct answers***

| No. | Questions and filters | Coding | categories |
| --- | --- | --- | --- |
| Q1 | School | |  |  |  | | --- | --- | --- | | PS………..1  JSS………..2  SSS……….3 |
| Q2 | Circle Sex of the respondents | Male  Female | ……………..1……………...2 |
| Q3 | How old are you in complete years? | 12 years old  13 years old  14 years old  15 years old  16 years old  17 years old  18 years old | ….…………..1  …..………….2  …...…………3  ……………...4  ..…………….5  ..…………….6  ..…………….7  ..…………….8 |
| Q4 | In what grade or form are you? | Standard 6  Standard 7  Form 1  Form 2  Form 3  Form 4  Form 5  Form 6 | ……………..1  ……………..2  …………….3  …………….4  ……………..6  ……………..7  ……………..8  ……………..9  …………….10 |
| Q5 | How would you describe your home? | One room  Two rooms  Three rooms  More than three | ………………1  ………………2  ……..……….3  ……..……….4 |

**First, we would like to know what you think about cigarette smoking**

| No. | Questions and Filters | Coding categories | | |
| --- | --- | --- | --- | --- |
| Q6 | Please say whether you believe or don’t believe each of the statements below. Please don’t leave any out. For each statement put an x in the box to tell us your view. | I believe this  (code 1) | I don’t believe this  (code 2) | I don’t know  (code 9) |
|  | 1. Smoking a cigarette would be harmful to my health |  |  |  |
|  | 1. Smoking cigarettes helps with stress |  |  |  |
|  | 1. Smoking cigarettes makes you stronger |  |  |  |
|  | 1. Smoking cigarettes is expensive |  |  |  |
|  | 1. Smoking makes you confident |  |  |  |
|  | 1. Smoking cigarettes is enjoyable |  |  |  |
|  | 1. Smoking cigarettes makes your teeth look yellow |  |  |  |
|  | 1. Smoking cigarettes makes you more intelligent |  |  |  |
| Q7 | Please write in your own words what you think about smoking cigarettes. |  | | |
| Q8 | Please say whether you believe or don’t believe each of the statements below. | I believe this  (code 1) | I don’t believe this  (code 2) | I don’t know  (code 9) |
|  | 1. Most people my age smoke tobacco |  |  |  |
|  | 1. Smoking cigarettes is cool |  |  |  |
|  | 1. Smokers are bad people |  |  |  |
|  | 1. Smoking helps you make friends |  |  |  |
|  | 1. People who smoke cigarettes are more popular |  |  |  |
|  | 1. There are cool people who do not smoke |  |  |  |
|  | 1. Girls who smoke cigarettes are more attractive to boys |  |  |  |
|  | 1. Boys who smoke cigarettes are more attractive to girls |  |  |  |
|  | 1. People my age feel pressure to try smoking cigarettes |  |  |  |
|  | 1. Trying smoking is part of growing up |  |  |  |
|  | 1. Tobacco companies are very bad |  |  |  |
|  | 1. It’s easy for someone my age to get cigarettes |  |  |  |
| Q9 | I think someone my age who smokes cigarettes looks:  For each statement circle to tell us your view. You can select as many options as you want. | Normal ........1  Grown up ........2  Cool ........3  Attractive ........4  Smart (well presented) ........5  Strong-willed ........6  Weak ........7  Stupid ........8  Boring ........9  Easily led ......10  *Other*  _____ | | |
| Q10 | I think someone my age who does not smoke cigarettes looks:  For each statement circle to tell us your view. You can select as many options as you want. | Normal …….1  Grown up ………2  Cool ………3  Attractive ……….4  Smart (well presented) ……….5  Strong-willed ………6  Weak ………..7  Stupid ………..8  Boring ………9  Easily led ………10  *Other _______* | | |
| Q11 | Do you feel that you could refuse a cigarette if a friend offered it to you? | Yes ……….1  No ……….2  I’m not sure ……….3 | | |
| Q12 | In the next 12 months, do you think you might smoke a cigarette? | Yes ……..1  No ………2  May be ………3 | | |

**Next, we would like to know what you have seen and heard about smoking**

| No. | Questions and Filters | Coding | Categories |
| --- | --- | --- | --- |
| Q13 | In the past two months, have you had a conversation with any adults about smoking? (You can choose more than one answer) | Yes they told me about how harmful it is  Yes we talked about how I feel about smoking  Yes they encouraged me to refuse cigarettes  Yes they encouraged me to try smoking  Yes they tried to sell me cigarettes  No | ..................1  ..................2  ..................3  ..................4  ……............5  ……............6 |
| Q14 | In the past two months, have you had a conversation with any of your friends about smoking? (You can choose more than one answer) | Yes we talked about how harmful it is  Yes we talked about how we feel about smoking  Yes we talked about how to refuse cigarettes  Yes they encouraged me to try smoking  Yes they tried to sell me cigarettes  No | ..................1  ……............2  ..................3  ..................4  ..................5  ..................6 |

| No. | Questions and Filters | Coding | Categories |
| --- | --- | --- | --- |
| Q15 | In the past month, have you seen or heard any messages on the radio, on TV, on posters or in magazines about the harms of smoking? | Yes lots  Yes some  No none | .……….1  ………..2 ..........3 |
| Q16 | In the past month, have you seen any advertisements for cigarettes or tobacco? | Yes lots  Yes some  No none | .............1  .............2  .............3 |
| Q17 | In the past month, have you seen anyone smoking in films, videos, TV or magazine pictures? | Yes lots  Yes some  No, not at all | .............1  .............2  .............3 |

**The next questions ask about whether you have ever tried tobacco.**

| No. | Questions and Filters | Coding | Categories | |
| --- | --- | --- | --- | --- |
| Q18 | Have you ever tried smoking cigarettes or any form of tobacco, even if it was only one or two puffs? | Yes  No | ……............1  ..................2 | |
| Q19 | How many times have you tried to smoke a cigarette, even just a puff?  Circle in the categories | Never  Just once  Two or three times  Lots of times | ………………..1  ………………..2  ..………………3  ..………………4 | |
| Q20 | How old were you when you first tried smoking?  *Write in the age when you first had a puff on a cigarette, or leave blank if you have never tried smoking.* | |  |  | | --- | --- | | Don’t remember ……………1 | |
| Q21 | Do you want to try a cigarette or even just a puff?  *Circle your answer in the categories section.* | Yes  No | …….…………1  …………….…2 | |
| Q22 | In the last month, on how many days did you smoke cigarettes?  *Circle the correct category for your answer.* | 0 days  1 or 2 days  3 to 5 days  6 to 9 days  10 to 19 days  20 to 29 days  Every day (all 30 days) | ..................1  ..................2  ..................3  ..................4  ..................5  ..................6  ..................7 | |
| Q23 | In the last two months, has anyone offered you a cigarette?  *Circle in the correct category* | Yes, someone my age  Yes, an adult  No | ..................1  ..................2  ..................3 | |
| Q24 | If someone offered you a cigarette, what did you do?  *Circle in the correct category* | I took it and smoked it  I took it and pretended to smoke it  I took it but didn’t smoke it  I didn’t take it  No one offered me | ………………..1  ………………..2  ………….…….3  …………..……4  …………..……5 | |
| Q25 | What would you say if you are offered a cigarette?  *Write in your answer* |  | | |
| Q26 | Have you ever tried any form of tobacco that isn’t smoked  (e.g. chewing tobacco, snuff, dip, kuber)?  *Circle in the categories for the correct answer.* | Yes  No | | …………1  …………2 |
| Q27 | Have you ever tried any other tobacco product?  If yes, what?  *Write your answer.* |  | |  |
| Q28 | Do any of your close friends smoke?  *Circle your answer in the categories section. You may choose more than one option.* | Yes most of my close friends smoke  Yes some of my close friends smoke  No, none of my close friends smoke | | …………..1  ……..……2  .………….3 |
| Q29 | Do any of your close family smoke?  *Circle your answer in the categories section. You may choose more than one option.* | Yes, my mother (or female guardian/care giver)  Yes, my father (or male guardian/care giver)  Yes, my older sister  Yes, my older brother  Yes, someone else in my close family  No, none of my close family | | …………..1  …………..2  …………..3  ….……….4…..………5  …..………5 |
| Q30 | Do you want smoke when you grow up?  *Circle the correct category for your answer.* | Yes  No  Don’t know | | …………..1  …………..2  ..…………9 |

**Thank you for your time**
